# CDK5RAP2 is a Wnt target gene and promotes stemness and progression of oral squamous cell carcinoma

**DOI:** 10.1038/s41419-023-05652-z

**Published:** 2023-02-11

**Authors:** Yuehong Shen, Yuling Chen, Yuntao Lin, Yicun Li, Pengfei Liu, Biru Zhang, Yufan Wang, King-Chi Chan, Nai-Ki Mak, Michael Kahn, Robert Z. Qi, Hongyu Yang

**Affiliations:** 1 Department of Oral and Maxillofacial Surgery, Stomatological Center, Peking University Shenzhen Hospital; Guangdong Provincial High-level Clinical Key Specialty; Guangdong Province Engineering Research Center of Oral Disease Diagnosis and Treatment; The Institute of Stomatology, Peking University Shenzhen Hospital, Shenzhen Peking University-The Hong Kong University of Science and Technology Medical Center, Guangdong, China; 2 Division of Life Science and State Key Laboratory of Molecular Neuroscience, The Hong Kong University of Science and Technology, Hong Kong, China; 3 Department of Stomatology, Shenzhen Luohu People’s Hospital, Guangdong, China; 4 Centre for PanorOmic Sciences, The University of Hong Kong, Hong Kong, China; 5 Department of Biology, Hong Kong Baptist University, Hong Kong, China; 6 Department of Molecular Medicine, City of Hope, Beckman Research Institute, Duarte, CA USA; 7 Bioscience and Biomedical Engineering Thrust, Systems Hub, The Hong Kong University of Science and Technology (Guangzhou), Guangdong, China

**Keywords:** Cancer stem cells, Oral cancer

## Abstract

In oral squamous cell carcinoma (OSCC), a highly aggressive and frequently lethal malignancy, the role and action mechanism of the microtubule regulatory protein CDK5RAP2 have not been fully understood. Here, we show that CDK5RAP2 is highly expressed in OSCC and its expression correlates with clinical stage and lymph node metastasis of the disease. The expression of CDK5RAP2 is regulated by the Wnt signaling pathway. Depletion of CDK5RAP2 inhibits the tumorigenesis and migration of OSCC cells and alters the OSCC cancer stem (-like) cell (CSC) signature. Notably, suppression of CDK5RAP2 expression disrupts spindle orientation during mitosis. Collectively, these results identify CDK5RAP2 as a potential CSC marker and reveal a mechanism that controls the CSC population in OSCC.

## Introduction

Head and neck squamous cell carcinoma (HNSCC) is a prevalent, malignant, and highly aggressive form of cancer that arises from the mucosal epithelium of the oral cavity, nasopharynx, oropharynx, larynx, and hypopharynx [1]. More than 90% of cancer cases in the head and neck region are oral squamous cell carcinoma (OSCC) [2]. The standard treatment for OSCC is surgical excision combined with radiotherapy and chemotherapy. However, the 5-year survival rate for advanced patients with OSCC remains low due to tumor recurrence, metastasis, and treatment resistance, which is at least partially driven by a specific population of cancer cells [3–5]. Recent evidence suggests the existence of a subpopulation of cancer cells with stem-like properties, known as cancer stem (-like) cells (CSCs), which have been isolated from most types of cancer. CSCs possess the ability to self-renew, thereby facilitating tumor growth, and contributing to tumor relapse and metastasis [6, 7]. Several stem cell markers, including ALDH1 [8, 9], SOX2 [10, 11], CD44 [12, 13], Notch1 [14, 15], CD133 [16], EZH2 [17], and CCND1 [18], have been identified in cancer cells, and these markers are considered to be the core regulatory proteins in CSCs that maintain their self-renewal properties. Targeting these CSC markers is emerging as an effective therapeutic strategy for impeding tumor progression. However, the underlying mechanisms regulating CSCs remain largely unknown.

CDK5RAP2 is a widely expressed protein whose loss-of-function mutations result in autosomal recessive primary microcephaly, a cell-cycle-dependent neural progenitor disorder that occurs during neurogenesis [19–21]. CDK5RAP2 is a centrosomal protein that interacts with the γ-tubulin ring complex (γTuRC), the principal microtubule nucleator in cells, to stimulate γTuRC-dependent microtubule nucleation [22, 23]. Here, we report that CDK5RAP2 is highly expressed in OSCC and its upregulation is associated with poor clinical outcomes, such as advanced stages of the disease and metastasis. Additionally, the expression of CDK5RAP2 is regulated by a signalling cascade called the canonical Wnt pathway, which is known to be involved in cancer development and metastasis [24–26]. Depletion of CDK5RAP2 in OSCC cells inhibits tumorigenesis and migration, and reduces the expression of markers associated with cancer stem cells. Overall, these findings suggest that CDK5RAP2 is not only a potential biomarker for progression in OSCC, but also a potential target for developing new therapies for this type of cancer.

## Materials and methods

### Patients and tissue samples

OSCC tissues and the paired normal tissues were obtained from 50 patients at the Department of Oral and Maxillofacial Surgery, Stomatological Center, Peking University Shenzhen Hospital (Shenzhen, China). The patients did not receive any cancer treatment, such as chemotherapy or radiotherapy, before surgery. The tissue specimens were snap-frozen in liquid nitrogen and then stored at −80 °C until use, or embedded in paraffin for use in immunohistochemistry (IHC) analysis. The cancer diagnoses and classification through pathological examination were based on the classification system of the World Health Organization. Written informed consent was obtained from all patients. This study was approved by the Ethics Committee of the Peking University Shenzhen Hospital. All experiments were performed in accordance with the principles of the Declaration of Helsinki.

### IHC analysis

IHC staining was performed as described [27]. Briefly, tissues were embedded in paraffin and dissected. The sections were subsequently stained according to the manufacturer’s instructions (MBX Biotechnologies, cat. no. KIT-9710, China). Images were captured under an upright fluorescence microscope (BX53, Olympus, Japan), and then a semi-quantitative method involving the use of Image J was applied to score expression levels and to calculate histoscores. Each section was automatically sorted according to the immunostaining intensity into one of four groups—high positive, positive, low positive, or negative—and the histoscores were calculated thus: Histoscore = (3 × percentage of high-positive stained area) + (2 × percentage of positive stained area) + (1 × percentage of low-positive stained area) + (0 × percentage of negative stained area). Histoscores ranged from 0 to 300.

### Cell culture

Human oral keratinocyte (HOK) cells were obtained from the cell bank of the Chinese Academy of Sciences (Shanghai, China). HSC-3 cells were purchased from the BeNa Culture Collection (BNCC341400, China); Cal27, RPE1, and HeLa cells were purchased from American Type Culture Collection (VA, USA) as the cell lines CRL-2095, CRL-4000, and CRM-CCL-2^TM^, respectively. HSC-3 and Cal27 are OSCC cell lines. HeLa, which is cervical cancer cell line, is a model commonly used for spindle orientation studies [28]. These cell lines were authenticated using short tandem repeat profiling analysis. All cell lines were confirmed to be free of mycoplasma contamination. All cell lines were cultured at 37 °C and 5% CO_2_ in a humidified atmosphere. HOK, HSC-3, Cal27 and HeLa cells were cultured in Dulbecco’s modified Eagle’s medium (DMEM; Gibco, CA, USA) containing 10% fetal bovine serum (FBS; Gibco) and 1% penicillin/streptomycin. RPE1 cells were cultured in 1:1 DMEM/F12 (Gibco) containing 10% FBS, 1% penicillin/streptomycin. Cell treatments with ICG-001 (Selleck, TX, USA) and Wnt3a (R&D Systems, MN, USA) were described in the appropriate figure legends.

### Antibodies

The following antibodies were purchased: for Western blotting, anti-β-catenin (1:5000; Abcam, cat. no. ab32572), anti-β-actin (1:5000; Bioss, cat. no. bs-0061R), anti-CDK5RAP2 (1:2000; Abcam, cat. no. ab70213), anti-survivin (1:2000; Abclonol, cat. no. A1551), anti-ALDH1 (1:1000; Cell Signaling Technology, cat. no. 54135), anti-Notch1 (1:1000, Abcam, cat. no. ab52627), anti-EZH2 (1:1000; Cell Signaling Technology, cat. no. 5246), anti-CCND1 (1:1000, Cell Signaling Technology, cat. no. 55506), and horseradish peroxidase-conjugated secondary antibodies (1:1000; Cell Signaling Technology, Inc.); for IHC analyses, anti-CDK5RAP2 (1:400; Abcam, cat. no. ab235893), anti-ALDH1 (1:50; Abcam, cat. no. ab52492), anti-SOX2 (1:100; Abcam, cat. no. Ab92494), anti-CD44 (1:4000; Abcam, cat. no. ab189524), anti-CD133 (1:1000; Abcam, cat. no. ab222782), anti-Notch1 (1:150; Abcam, cat. no. ab52627), anti-EZH2 (1:200; Cell Signaling Technology, cat. no. 5246), and anti-CCND1 (1:200; ABclonal, cat. no. A19038); and for immunofluorescence analyses, anti-α-tubulin (1:500, YL1/2; Santa Cruz Biotechnology, cat. no. sc-53029), anti-γ-tubulin (1:1000, GTU88; Sigma-Aldrich, cat. no. T5326), and Alexa Fluor-conjugated secondary antibodies (1:500; Thermo Fisher Scientific).

### Chromatin immunoprecipitation (ChIP) assay

ChIP assays were performed by using the SimpleChIP® Plus Sonication Chromatin IP Kit (Cell Signaling Technology, cat. no. 56383). Briefly, cells treated with or without ICG-001 were grown to 80% confluence and fixed with 1% formaldehyde. After stopping the fixation process by adding glycine to a final concentration of 0.125 M, the cells were lysed and centrifuged at 300 × *g*, and the obtained chromatin was re-suspended and then sheared through sonication into 500 bp DNA-protein fragments. The cell lysates were incubated with antibodies against CREB-binding protein (CBP, the target) or IgG (negative control), and Protein A beads were used to capture the antibody complexes. Lastly, the precipitated DNA was purified using the purification columns and qRT-PCR was used to analyze the enrichment of DNA fragments. The following primer set was used to detect enrichment of the DNA fragments from the *cdk5rap2* promoter region: CDK5RAP2-F, 5′-CGACAGAGGCACCATTTCAA-3′; CDK5RAP2-R, 5′- GCCAGCAAGAGGAAAGGACT -3′.

### Luciferase assay

The *cdk5rap2* promoter region was analyzed using the Ensembl website (https://asia.ensembl.org/index.html). According to the predicted CBP-binding sites, three promoter fragments, BS1-5 (1268 bp), BS1-3 (893 bp), and BS1-2 (670 bp), were amplified using these primers: BS1-5 Forward, 5′-GAAGATCTTTCATCGGGCTCTCTCACAA-3′; BS1-3 Forward, 5′-GAAGATCTCGACAGAGGCACCATTTCAA-3′; BS1-2 Forward, 5′-GAAGATCTGGAGTGGGAGTGCTGTTGAC-3′; Promoter Reverse, 5′-CCAAGCTTGGCTACAGAGGTGGCGA-3′. The obtained DNA fragments were cloned into the luciferase reporter vector pGL3-basic (Promega). The pGL3 constructs carrying *cdk5rap2* promoter fragments were co-transfected with pRL-TK (containing the Renilla luciferase construct; Promega) into cells, which were treated with 25 μM ICG-001 or DMSO at 24 h post-transfection, and after another 24 h, luciferase activity was measured using the Dual-Luciferase® Reporter Assay System (Promega) and quantified as relative luciferase units (RLU): RLU = (firefly luciferase intensity/Renilla luciferase intensity) × 100%.

### Generation of stable CDK5RAP2-knockdown cells

Packaged lentivirus constructs (pHBLV-U6-MCS-CMV-ZsGreen-PGK-PURO) encoding CDK5RAP2 shRNA (TGGAAGATCTCCTAACTAA) or control shRNA were generated by Hanbio (Shanghai, China). Cells were seeded into 24-well plates and allowed to grow to 30–50% confluence, and then culture media mixed with shRNA lentiviral particles were added to the wells and the plates were incubated for 24 h. To select for infected cells, the cells were cultured in medium containing 10 μg/mL puromycin dihydrochloride (MedChemExpress) for ~72 h, after which the selected stable cells were cultured in medium supplemented with 0.5 μg/mL puromycin dihydrochloride. CDK5RAP2 knockdown efficiency was determined through Western blotting.

### Cellular senescence assay

HSC-3 and Cal27 cells were seeded in 6-well plates (1 × 10^5^ cells/well) and cultured for 24 h. The cells were examined for senescence by using the β-galactosidase staining kit (Beyotime, China) according to the manufacturer’s instruction. Images were acquired under a microscope (Leica, Germany).

### Colony formation assay

shRNA-carrying stable cell lines were seeded at low densities. After culturing for 2-3 weeks, the cells were washed with phosphate-buffered saline (PBS), fixed with 4% paraformaldehyde, and stained with 0.1% crystal violet (Sigma, C3886-25G). The formed colonies were imaged using a Nikon microscope (Nikon, Japan).

### In vivo tumorigenicity assay

Female BALB/c athymic nude mice aged 4–5 weeks old were purchased from GemPharmatech Co., Ltd. The animal study was approved by the Experimental Animal Ethics Committee of the Shenzhen PKU-HKUST Medical Center. The mice were randomly and blindly divided into two groups. Four mice were in each experimental group. shRNA-carrying stable lines of Cal27 cells (4 × 10^6^/100 μL) were subcutaneously injected into the flank area of the nude mice, and tumor growth and mouse weight were then monitored every 4 days. After 4 weeks, the mice were sacrificed and tumors were weighed. The isolated tumors were dissected for IHC analysis.

### Wound-healing assay

shRNA-carrying stable cell lines were seeded into 6-well plates and cultured until a confluent monolayer was formed, a pipette tip was used to make a scratch on the cell monolayer. The cells were washed thrice with PBS and incubated with fresh medium containing 1% FBS to induce migration, and images were acquired at various time points.

### Transwell migration assay

shRNA-carrying stable cell lines suspended in DMEM lacking FBS were added into the upper chamber of transwell chambers (Corning, NY, USA), and medium containing 10% FBS was added to the lower chamber. After incubation for 48 h, the cells in the upper chamber were wiped off, and the cells in the lower chamber were fixed with 4% paraformaldehyde and stained with 0.1% crystal violet. Images were acquired under a microscope and analyzed by using image J.

### Tumorsphere formation assay

shRNA-carrying stable cells were suspended and cultured at 5000 cells/well in 6-well ultra-low-attachment plates (Corning), and the culture medium was serum-free and supplemented with basic fibroblast growth factor and epidermal growth factor (20 ng/mL each; Novoprotein, China) and N-2 supplement (Gibco, CA, USA). After 7–10 days, we counted the tumorspheres (diameter >50 µm) of Cal27 and HSC-3 cells.

### Bioinformatics

The correlation coefficient of RNA expression levels between CDK5RAP2 and known HNSCC stem cell markers was calculated using the HNSCC dataset from The Cancer Genome Atlas (TCGA). R package Corrplot (version 0.90) was used to plot the correlation coefficients. Dotplots of the detailed expression values were drawn using R package ggplot2 (version 4.0.5).

### Spindle orientation assay

Spindle angles were measured as described in a previous report [29]. Cells were seeded on fibronectin-coated coverslips and synchronized by the treatment of 100 ng/mL nocodazole (Sigma, MO, USA; M1404) for 14 h. After recovery for 30 min, the cells were fixed with methanol for 10 min at −20 °C and then stained with anti-α-tubulin and anti-γ-tubulin antibodies. Hoechst 33258 (Sigma, MO, USA; 94403) was used at 1 μg/mL to stain nuclei. Fluorescence images were acquired using a confocal microscope (STELLARIS 5, Leica, Germany), and Z-stack scans of metaphase cells were obtained (section thickness: 0.3 μm). The spindle angle (α^ο^) was calculated using the following inverse trigonometric function: α^ο^ = tan^-1^ (Z/X) (Z, vertical distance between two spindle poles; X, horizontal distance between two spindle poles).

### Statistics and reproducibility

All results were analyzed using Image J and GraphPad Prism 5.0 software. The data presented in graphs are means ± SEM or SD, as indicated in figure legends. At least three independent experiments were performed for each condition. Cumulative survival was determined via the Kaplan-Meier method. Statistical significance was set at *P* < 0.05.

## Results

### CDK5RAP2 expression is increased in human OSCC

**Fig. 1:**
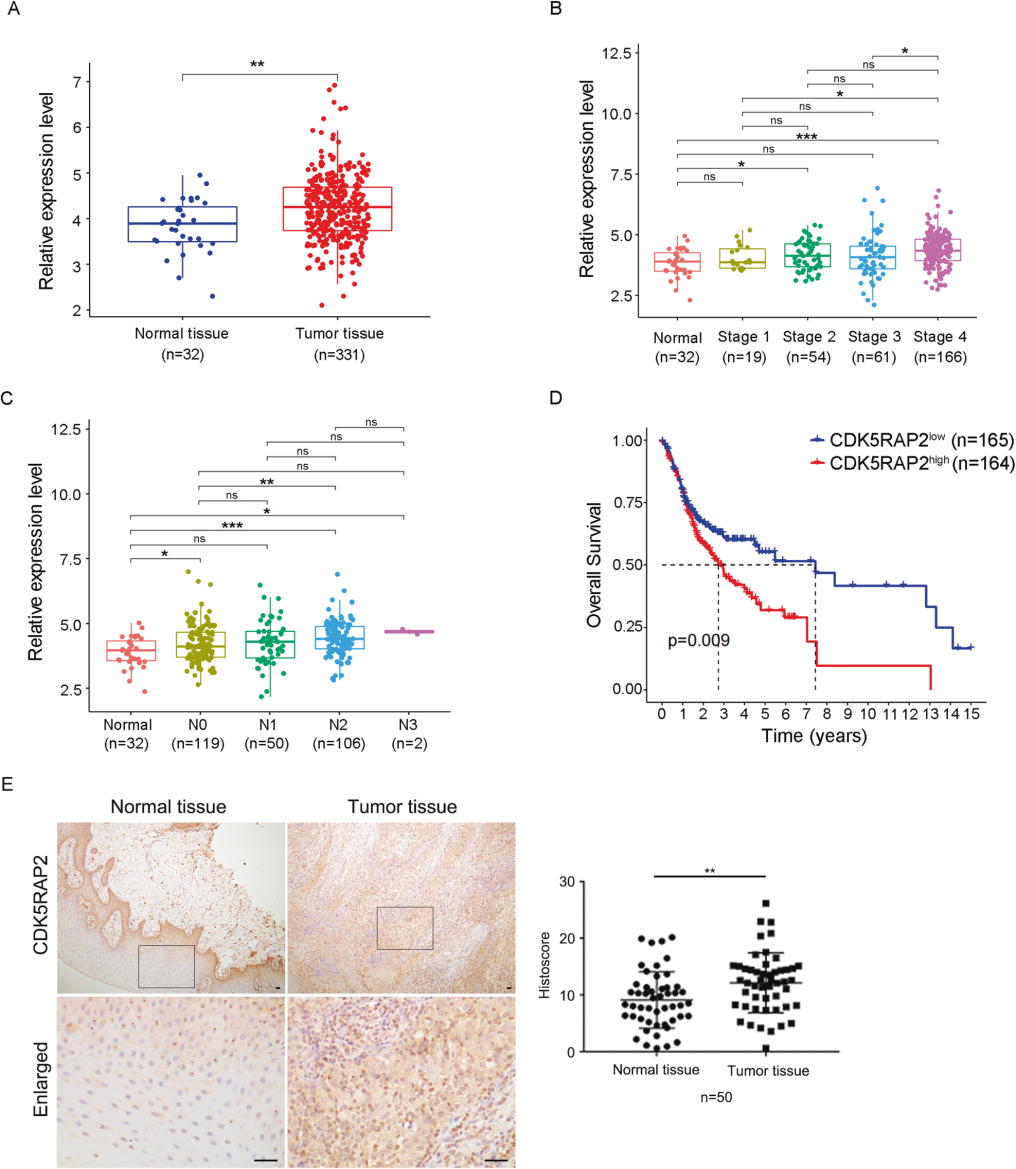
CDK5RAP2 is upregulated in OSCC. **A**–**C** CDK5RAP2 expression in OSCC according to sample types **A**, cancer stages **B**, and nodal metastasis status **C**. **D** Kaplan–Meier survival analysis of TCGA-OSCC cohorts grouped by CDK5RAP2 expression level. **E** IHC staining of CDK5RAP2 in OSCC. Rectangular area is enlarged. CDK5RAP2 expression was quantified using Image J (*n* = 50). Scale bars, 20 μm. Data are presented as means ± SEM; *ns* no significance, ^*^P < 0.05, ^**^P < 0.01, ^***^P < 0.001.

To determine the expression of CDK5RAP2 in OSCC, we analyzed the OSCC dataset from TCGA, with fold-change > 2.0 and *P* < 0.05 being used as the cutoff values in the expression analysis. The results showed that CDK5RAP2 expression was significantly higher in tumor tissues than in normal tissues (Fig. 1A). To further investigate the correlation between CDK5RAP2 expression and OSCC clinical characteristics, we analyzed the data based on cancer stages and lymph node metastasis status. Our analysis revealed that CDK5RAP2 expression was significantly increased in patients with higher clinical stages and higher number of lymph node metastasis (Fig. 1B, C). Furthermore, we performed survival analysis using the Kaplan–Meier method and found that the high expression of CDK5RAP2 was correlated with poor survival outcomes (Fig. 1D).

To validate our findings from the OSCC dataset from TCGA, we conducted an additional study using a cohort of 50 pairs of OSCC and adjacent normal tissue samples. We performed IHC staining, and the results confirmed that CDK5RAP2 expression was increased in the tumor tissues compared to the normal tissues (Fig. 1E). Furthermore, to investigate the correlation between CDK5RAP2 expression and the clinicopathological features of OSCC patients, we examined various clinical characteristics, such as gender, age, tumor size, differentiation grade, clinical stage, and lymph node metastasis. The analysis of these characteristics revealed that CDK5RAP2 expression was increased in human OSCC and closely related to tumor stage and metastasis (Table 1). These results were consistent with the findings from the TCGA dataset analysis.

**Table 1:** Clinicopathologic features of OSCC patients.

Characteristics	Number of cases (*n* = 50)	Relative CDK5RAP2 histoscore (Mean ± SD)	*P* value
Gender			0.5626
Male	40	1.625 ± 0.794	
Female	10	1.448 ± 1.104	
Age (years)			0.1229
≥60	18	1.839 ± 1.132	
<60	32	1.449 ± 0.629	
Tumor size (cm)			0.7194
≥4	15	1.657 ± 0.925	
<4	35	1.561 ± 0.835	
Differentiation grade			0.4359
Well	36	1.649 ± 0.969	
Moderately and Poorly	14	1.437 ± 0.440	
Clinical stage			0.0269^*^
0&I+II+III	27	1.345 ± 0.480	
IV	23	1.877 ± 1.094	
Lymph node metastasis			0.0328^*^
Yes	27	1.826 ± 1.030	
No	23	1.312 ± 0.475	

^*^P < 0.05.

### CDK5RAP2 expression is regulated by Wnt signaling pathway

**Fig. 2:**
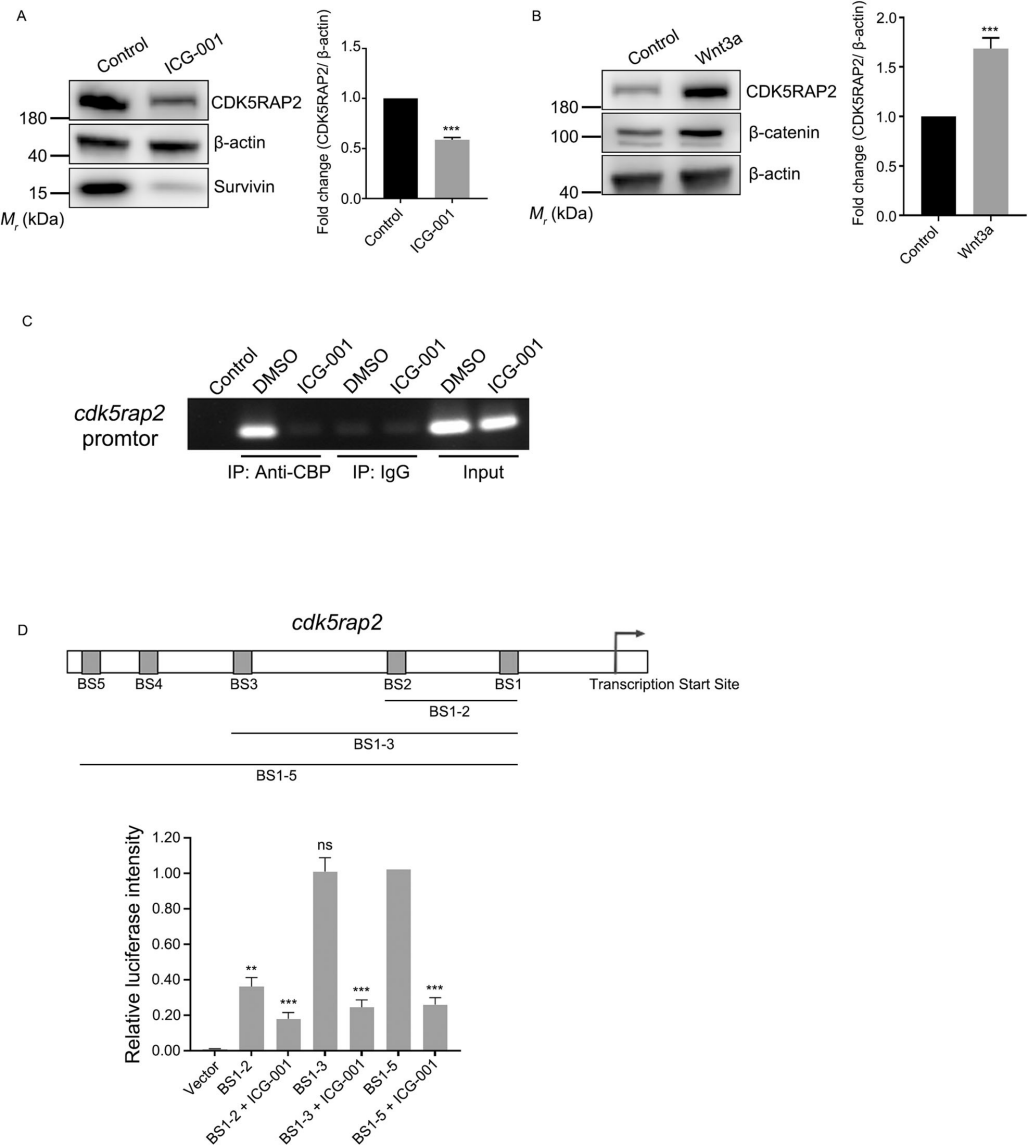
CDK5RAP2 expression is regulated by the Wnt signaling pathway. **A**, **B** HOK cells were treated with 25 μM ICG-001 **A** or 100 ng/mL Wnt3a **B** for 24 h and then cell lysates were immunoblotted with the indicated antibodies. CDK5RAP2 protein levels were quantified and normalized to the β-actin protein levels. **C** ChIP assay was performed using rabbit anti-CBP antibody or normal rabbit IgG. RPE1 cells were treated with DMSO as mock treatment or 25 μM ICG-001. **D** Schematic of predicted CBP-binding sites in the *cdk5rap2* promoter region. Binding between CBP and the *cdk5rap2* promoter was determined using a dual-luciferase reporter assay. RPE1 cells transfected with distinct promotor fragments were treated with or without ICG-001. Firefly luciferase intensity was normalized relative to *Renilla* luciferase intensity. Data are presented as means ± SD; *ns* no significance, ^**^P < 0.01, ^***^P < 0.001, two-tailed *Student*’s *t*-test.

Considering that the Wnt signaling pathway plays essential roles in the development of several types of cancer, including HNSCC [30–32], we treated cells with ICG-001, a small-molecule inhibitor that specifically blocks the interaction between the CBP and β-catenin, thus acts as a selective antagonist of the canonical Wnt/β-catenin signaling pathway [33]. Western blotting results showed that the level of survivin was decreased after ICG-001 treatment, which is consistent with a previous study [34]. Furthermore, the level of CDK5RAP2 was significantly decreased after the inhibitor treatment (Fig. 2A). Notably, in cells treated with ICG-001, the CSC markers, such as ALDH1, EZH2, Notch1, and CCND1, were significantly decreased (Supplementary Fig. 1). To further investigate the effect of Wnt signaling on CDK5RAP2, we treated cells with Wnt3a, a secreted protein that activates the Wnt/β-catenin signaling pathway [35, 36]. Western blotting results showed that the levels of CDK5RAP2 were increased after the Wnt3a treatment (Fig. 2B).

To further confirm the regulation of CDK5RAP2 by the Wnt/β-catenin signaling pathway, we performed ChIP assays to analyze the interaction between CBP and the *cdk5rap2* promoter. We found that CBP interacted with the *cdk5rap2* promoter, and that ICG-001 treatment abolished the binding (Fig. 2C). To assess promoter activity, we cloned DNA fragments containing the predicted CBP-binding sites in the *cdk5rap2* promoter region and performed a luciferase assay. The luciferase activity was low in cells transfected with the plasmid carrying both binding sites 1 and 2, but notably increased when the plasmid included binding site 3 (Fig. 2D). Accordingly, after ICG-001 treatment, the luciferase activity was decreased even in cells transfected with the plasmid carrying all 5 predicted CBP-binding sites (Fig. 2D). Collectively, these results showed that CDK5RAP2 expression is regulated by the Wnt/β-catenin signaling pathway.

### CDK5RAP2 downregulation inhibits the tumorigenesis and migration of OSCC cells

**Fig. 3:**
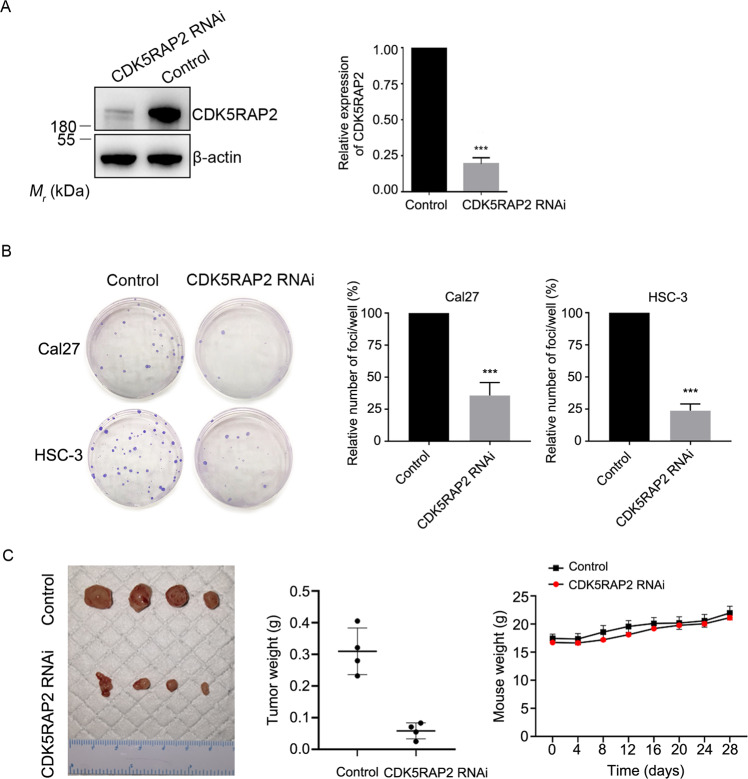
CDK5RAP2 downregulation inhibits OSCC tumorigenesis. **A** Cal27 cells were infected with a recombinant lentivirus containing CDK5RAP2 shRNA (CDK5RAP2 RNAi) or negative-control shRNA (Control), and then lysates of stable cells were immunoblotted with the indicated antibodies. **B** Colony-formation assay was performed using control and stable CDK5RAP2-knockdown cells. Colonies in each well were counted. **C** shRNA-carrying lines of Cal27 cells were injected subcutaneously into nude mice. The image shows isolated tumors. Mice were weighed every 4 days after injection, and the isolated tumors were weighed. Data are presented as means ± SD; ^***^P < 0.001, two-tailed *Student*’s *t*-test.

To investigate CDK5RAP2 function in carcinogenesis of OSCC cells, we suppressed CDK5RAP2 expression using an shRNA-carrying recombinant lentivirus. In the stable cells selected after infection, CDK5RAP2 knockdown efficiency was ~80% (Fig. 3A). These cells did not show any obvious cellular senescence when stained using the β-galactosidase staining kit (Supplementary Fig. 2). We then performed colony-formation assays to evaluate cell growth and found that fewer colonies were formed by CDK5RAP2-knockdown cells compared to control cells (Fig. 3B). To examine the effect of CDK5RAP2 depletion on carcinogenesis in vivo, we subcutaneously injected stable cells carrying the control or CDK5RAP2 shRNA into BALB/c athymic mice. Although the average weight of the mice was similar in the two groups, the average weight of the tumor generated by CDK5RAP2-knockdown cells was considerably decreased compared to that in the control group (Fig. 3C). These results reveal that the knockdown of CDK5RAP2 interferes with tumorigenesis.

**Fig. 4:**
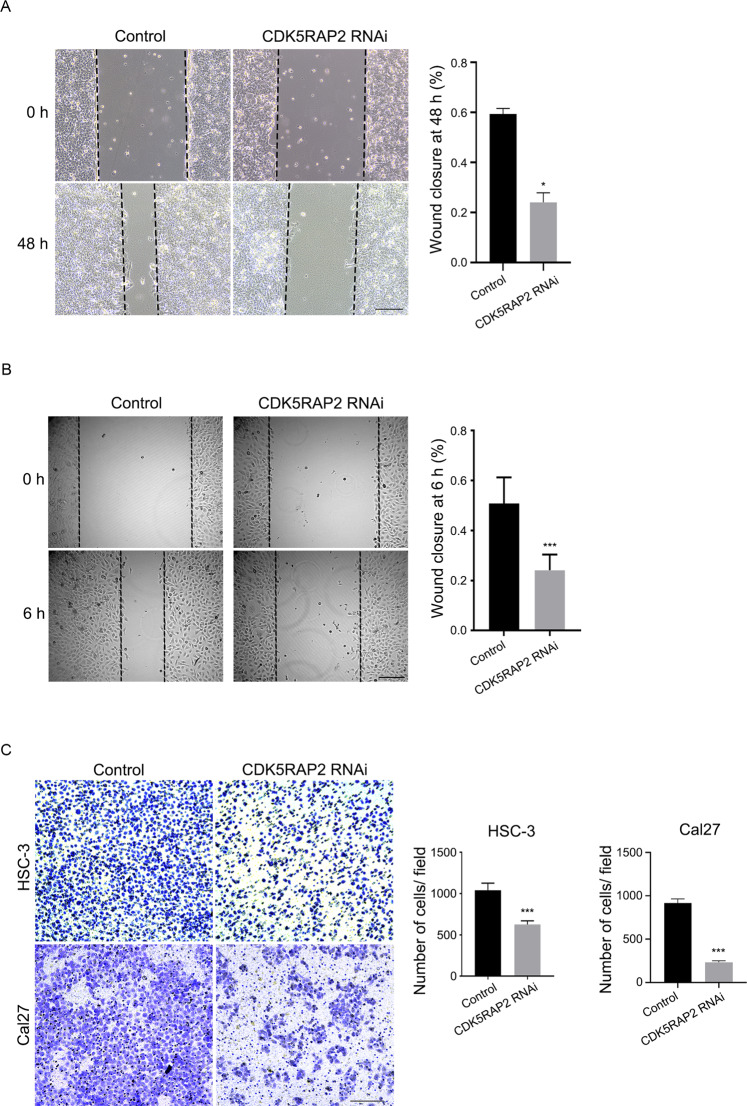
CDK5RAP2 downregulation inhibits OSCC cell migration. **A**, **B** Wound-healing assay was performed using Cal27 **A** and HSC-3 **B** stable cells. Representative images are shown. Ratios of wound closure at 48 h or 6 h were quantified. **C** Transwell migration assay was performed using stable cells. Representative images are shown. The migrated cell number per field was quantified. Data are presented as means ± SD; ^*^P < 0.05, ^***^P < 0.001, two-tailed *Student*’s *t*-test. Scale bars, 200 μm.

We utilized the wound-healing and transwell assays to evaluate the impact of CDK5RAP2 knockdown on cell migration. Notably, depletion of CDK5RAP2 resulted in reduced cell migration in both the wound-healing assay (Fig. 4A, B) and the lower chamber of the transwell assay (Fig. 4C). Collectively, these findings indicate that knocking down CDK5RAP2 impedes the migration of OSCC cells and tumorigenesis in vivo.

### CDK5RAP2 attenuates the stemness of OSCC cells and regulates spindle orientation

**Fig. 5:**
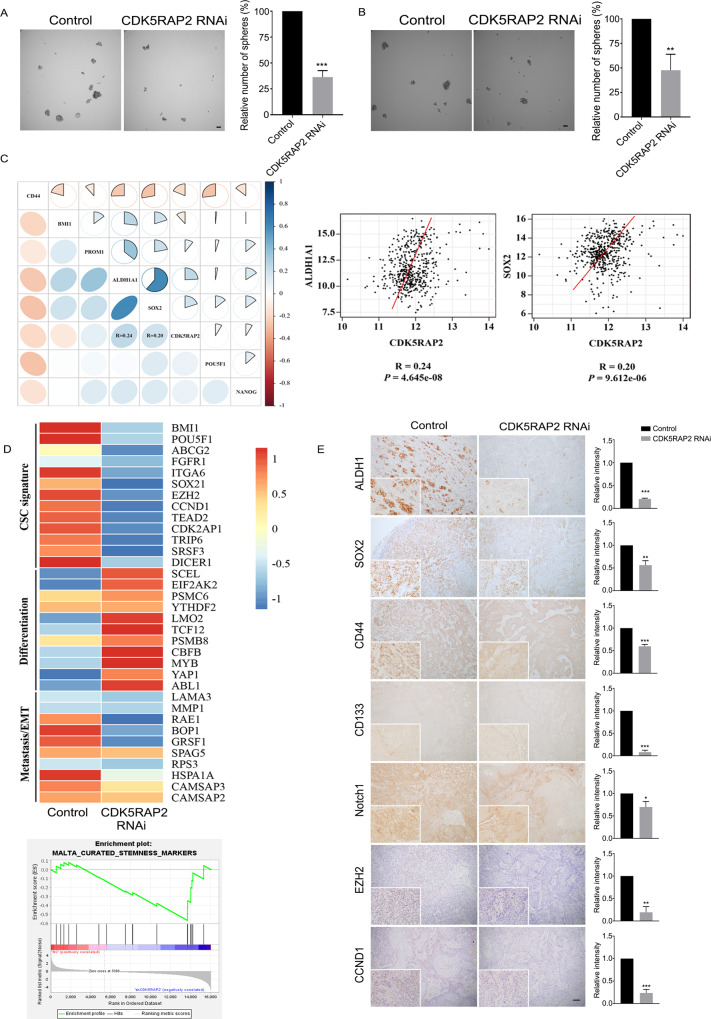
CDK5RAP2 knockdown suppresses stemness of OSCC cells. **A**, **B** Equal numbers of control and CDK5RAP2-knockdown stable Cal27 cells **A** or HSC-3 cells **B** were seeded into ultra-low-attachment plates and incubated for 8 days, after which the spheres (diameter > 50 μm) were counted. Scale bars, 50 μm. **C** Correlation between CDK5RAP2 and HNSCC CSC markers. **D** Heatmap presenting gene-expression profiles of control and stable CDK5RAP2-knockdown cells. Gene expression was measured using RNA-sequencing, and GSEA was performed. **E** Control and stable CDK5RAP2-knockdown cells were inoculated subcutaneously into nude mice. The panel shows representative images of tumor sections stained with antibodies against ALDH1, SOX2, CD44, CD133, Notch1, EZH2, and CCND1. Image J was used to analyze the relative expression intensities of indicated CSC markers from isolated xenograft tumors. Scale bar, 100 μm. All data are presented as means ± SD; ^*^P < 0.05, ^**^P < 0.01, ^***^P < 0.001, two-tailed *Student*’s *t*-test.

To further understand the mechanism by which CDK5RAP2 is involved in the growth and migration of OSCC cells, we tested cell stemness by performing the sphere-formation assay. After culturing CDK5RAP2-knockdown and control cells on culture medium for 8 days, we found that spheres formed by the knockdown cells were not only fewer in number but also smaller in size, as compared to those formed by the control cells (Fig. 5A, B). We also investigated the expression of several CSC markers in CDK5RAP2-knockdown and control cells by Western blotting. The results showed that the knockdown of CDK5RAP2 significantly decreased the levels of CSC markers such as ALDH1, EZH2, Notch1 and CCND1 (Supplementary Fig. 3). In addition, online bioinformatics analyses revealed a correlation between CDK5RAP2 expression and CSC markers in HNSCC (Fig. 5C).

To further investigate the effect of CDK5RAP2 knockdown on gene expression, we performed RNA-sequencing of CDK5RAP2-knockdown and control cells and compared the gene-expression profiles. Relative to control cells, CDK5RAP2-knockdown cells showed a > 2-fold expression upregulation and downregulation of 96 and 802 genes, respectively. The results of heatmap analysis indicated that certain genes associated with CSC signature and epithelial-mesenchymal transition (EMT) or metastasis were downregulated in CDK5RAP2 knockdown cells, whereas genes associated with differentiation were upregulated (Fig. 5D). The results of gene set enrichment analysis (GSEA) also revealed that the stemness signature was significantly diminished in CDK5RAP2-knockdown cells (Fig. 5D).

To confirm these findings in vivo, we again used the tumorigenesis assay to examine the expression of stemness markers in tumors. IHC staining of tumor sections revealed that the levels of ALDH1, CD44, CD133, SOX2, Notch1, EZH2 and CCND1 were downregulated in CDK5RAP2-knockdown tumors (Fig. 5E). These lines of evidence indicate that CDK5RAP2 regulates the stemness signature of OSCC cells.

**Fig. 6:**
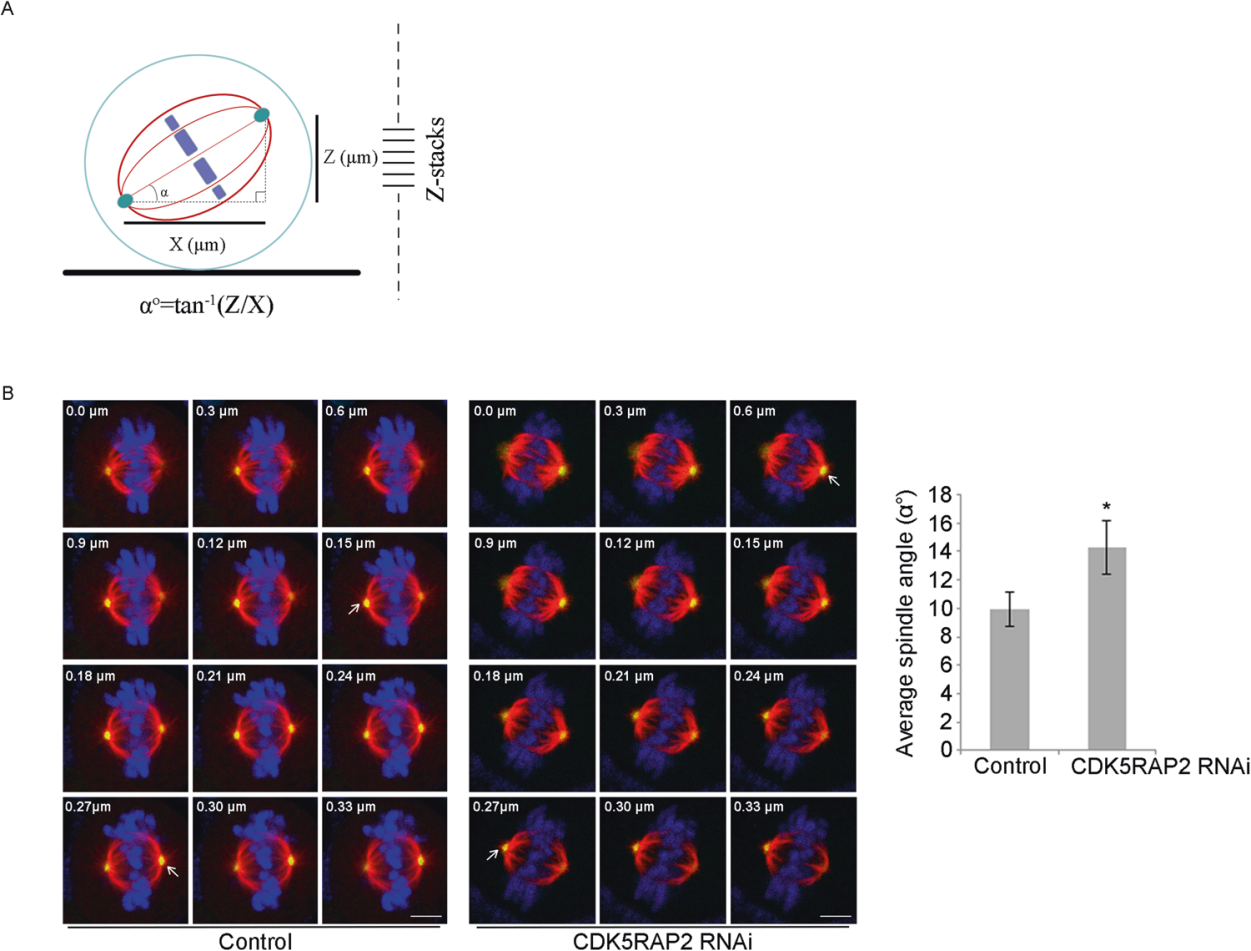
CDK5RAP2 regulates spindle orientation. **A** Schematic depicting calculation of spindle angles. The horizontal distance (X μm) and the vertical distance (Z μm) between two spindle poles of metaphase cells were measured by acquiring Z-stack images. Spindle angle (α^ο^), the angle between the spindle axis and the substrate plane, was calculated using an inverse trigonometric function. **B** Representative Z-stack images of metaphase cells. HeLa cells were fixed and immunostained with anti-α-tubulin and anti-γ-tubulin antibodies. White arrows indicate the focal planes of two spindle poles in two different sections. Scale bars, 5 μm. The spindle angle of HeLa cells after control or CDK5RAP2 knockdown was quantified. Data are presented as means ± SD; ^*^P < 0.05, two-tailed *Student*’s *t*-test.

Stem cells have the ability to undergo symmetric and asymmetric cell division, and the mode of division is determined by the orientation of mitotic spindles [37]. CDK5RAP2 plays critical roles in microtubule nucleation and organization, and its depletion causes mitotic spindle defects, including the lack of astral microtubules [22]. Because astral microtubules are crucial for spindle anchoring and orientation, we measured spindle angles in mitotic cells in which CDK5RAP2 expression was knocked down. In nonpolarized adherent cells, the spindles lie parallel to the substrate plane, an orientation that depends on integrin-mediated cell-substrate adhesion, and the spindle angle is the angle between the substrate plane and the spindle pole axis (Fig. 6A). Under a confocal microscope, we acquired Z-stack images of metaphase cells and used γ-tubulin immunostaining to visualize spindle poles (Fig. 6B). Our measurements revealed that CDK5RAP2 depletion significantly increased the spindle angle compared to that in control cells (Fig. 6B). These results indicate that CDK5RAP2 is involved in the control of spindle orientation.

## Discussion

CDK5RAP2 has previously been identified as a key player in the organization of microtubules in cells, acting as a stimulator of the γ-tubulin ring complex, which is a crucial microtubule nucleator required for microtubule organization [23]. The RNA transcript of CDK5RAP2 has been detected in all human tissues examined [19]. Furthermore, CDK5RAP2 interacts with microtubule plus-end binding protein EB1, thus regulating microtubule dynamics and cell motility [38–40]. In this study, we have uncovered a previously unknown function of CDK5RAP2 in OSCC. We have shown that: (1) CDK5RAP2 expression is abnormally high in OSCC tissues as compared to adjacent normal tissues, as revealed by IHC staining and TCGA-OSCC data analysis; (2) CDK5RAP2 expression is regulated by the Wnt signaling pathway; (3) CDK5RAP2 knockdown inhibits the tumorigenesis and migration of OSCC cells; and (4) CDK5RAP2 is found to be involved in maintaining the CSC signature of OSCC cells and in the regulation of spindle orientation.

CSCs are widely suggested to play a critical role during tumor initiation and progression, and CSCs thus hold critical clinical implications [41]. In 2007, Prince et al. reported that a CD44 + population of HNSCC cells exhibited a higher tumor-initiating ability relative to CD44- cells [12], and since then, CD44 has commonly been used as a CSC marker in HNSCC. Moreover, high activity of ALDH, which detoxifies aldehydes and oxidizes retinoic acid, is regarded as one of the major features of CSCs in HNSCC. ALDH1 is typically expressed at high levels in HNSCC primary tumors and cell lines [8, 42]. In addition to the aforementioned cell-surface marker and enzyme activity, proteins such as SOX2, which regulate CSC features, have also been identified as CSC markers [10, 11]. In this study, we have identified CDK5RAP2 as a potential CSC marker in OSCC. The results of TCGA dataset analysis showed that CDK5RAP2 was highly expressed in OSCC and significantly correlated with clinical stage and lymph node metastasis status. Importantly, manipulation of CDK5RAP2 expression regulated the CSC properties of OSCC cells, including migration, sphere formation, and tumorigenesis. Thus, our study suggests that CDK5RAP2 is a potential CSC marker that regulates CSC functions. It is also worth noting that several widely recognized CSC markers in HNSCC were downregulated after CDK5RAP2 depletion, indicating that CDK5RAP2 is required for maintaining the CSC population. This is reminiscent of the role of CDK5RAP2 in the maintenance of progenitor pools [21, 43].

CSCs play a significant role in cancer metastasis and drug resistance, and the size of CSC populations is closely linked to spindle orientation, which determines whether cell division occurs in a symmetric or asymmetric mode [44–46]. CDK5RAP2 is a crucial regulator of microtubule organization during the cell cycle, and CDK5RAP2 depletion results in diminished astral microtubules [22]. Astral microtubules connect the spindle poles to the cell cortex and thus control spindle orientation [47]. Our research found that knocking down CDK5RAP2 expression affects the spindle angle during mitosis and thus leads to spindle misorientation, and further that CDK5RAP2 depletion in OSCC cells alters their CSC signature, which is observed as, for example, a reduction in tumorsphere formation and downregulation of CSC markers. Our results show that CDK5RAP2 play critical roles in CSC functions in OSCC.

The Wnt signaling pathway is a key player in several cellular processes, including cell proliferation, differentiation, motility, and stemness maintenance [25]. Previous studies showed that Wnt3a plays a role in maintaining pluripotency in embryonic stem cells and orienting asymmetric stem cell division [48, 49]. Wnt3a is also highly expressed in HNSCC and HNSCC-derived cell lines, and the Wnt signaling pathway is crucial in maintaining the CSC signature of HNSCC [50–52]. Accordingly, treatment with Wnt activators or inhibitors alters the CSC proliferation rate [53, 54]. Furthermore, besides affecting CSC proliferation, activation of the Wnt signaling pathway increases the sphere-formation properties of HNSCC CSCs [55, 56]. Our research found that CDK5RAP2 expression is regulated by the Wnt signaling pathway through the association of CBP, a co-transcriptional factor of β-catenin, with the *cdk5rap2* promoter region, which modulates the expression of CDK5RAP2. This suggests that CDK5RAP2 is a new downstream target in the Wnt signaling pathway that regulates CSC properties.

Our research has found that CDK5RAP2 expression is increased in OSCC and is regulated by the Wnt signaling pathway. Importantly, suppression of CDK5RAP2 expression leads to a loss of control of spindle orientation and effectively inhibits the progression and alters the CSC signature of OSCC. These findings not only provide insight into the mechanisms by which CDK5RAP2 regulates OSCC progression, but also identify CDK5RAP2 as a potential CSC marker and therapeutic target for OSCC.

## Supplementary information

Supplementary Figures

Original Western blots of Figures

Author Contribution Statement

## Data Availability

The RNA sequencing data has been deposited in the NCBI BioProject database under the accession number PRJNA915333. All data and materials are available from the authors on request. Uncropped blots are shown in the Original Western blots of Figures.
