# Supplementary material for: CDK5RAP2 is a Wnt target gene and promotes stemness and progression of oral squamous cell carcinoma
Source: Cell Death Dis. 2023 Feb 11;14(2):107. doi: 10.1038/s41419-023-05652-z (PMC9922250; doi:10.1038/s41419-023-05652-z)
Supplement: Supplementary file 1 — Supplementary Figures [file 41419_2023_5652_MOESM1_ESM.pdf]

Supplementary Figure 1

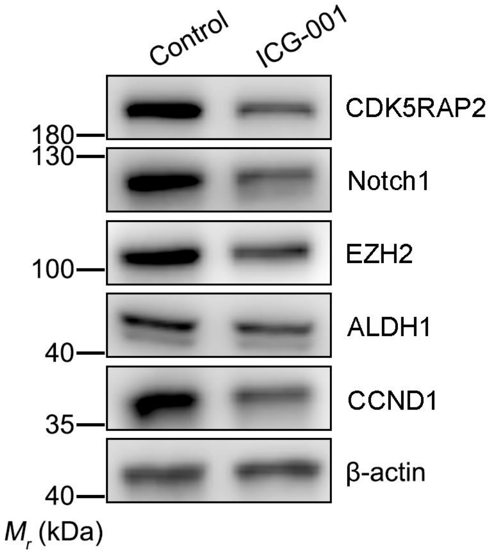

Supplementary Figure 1. Downregulation of CSC markers by treatment with ICG-001. HOK cells were treated with 25 µM ICG-001 for 24 h and cell lysates were then immunoblotted with the indicated antibodies.

Supplementary Figure 2

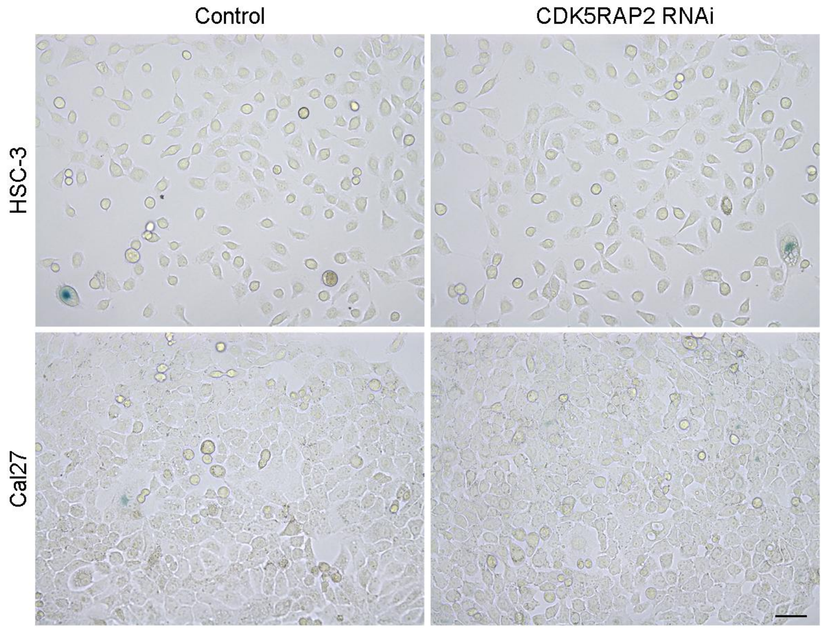

Supplementary Figure 2. Knockdown of CDK5RAP2 does not induce cellular senescence in OSCC cells. Cellular senescence was determined by β-galactosidase staining using control and stable CDK5RAP2-knockdown Cal27 and HSC-3 cells. Shown are representative images. Scale bar, 50 µm.

Supplementary Figure 3

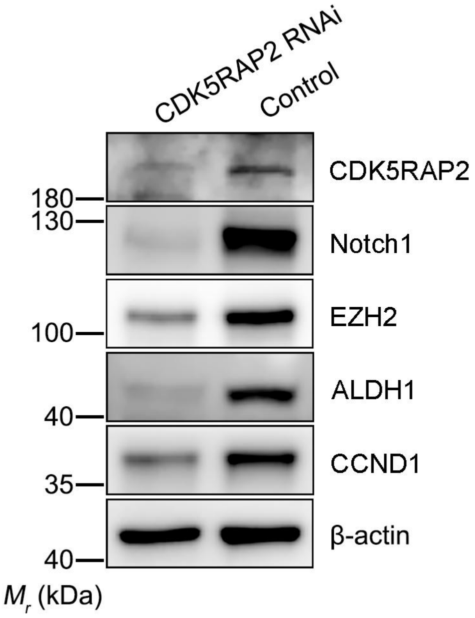

Supplementary Figure 3. Downregulation of CSC markers by CDK5RAP2 knockdown. Control and stable CDK5RAP2-knockdown Cal27 cells were lysed and immunoblotted for CSC markers.
