# Supplementary material for: CDK5RAP2 is a Wnt target gene and promotes stemness and progression of oral squamous cell carcinoma
Source: Cell Death Dis. 2023 Feb 11;14(2):107. doi: 10.1038/s41419-023-05652-z (PMC9922250; doi:10.1038/s41419-023-05652-z)

Original Western blots of Figure 2

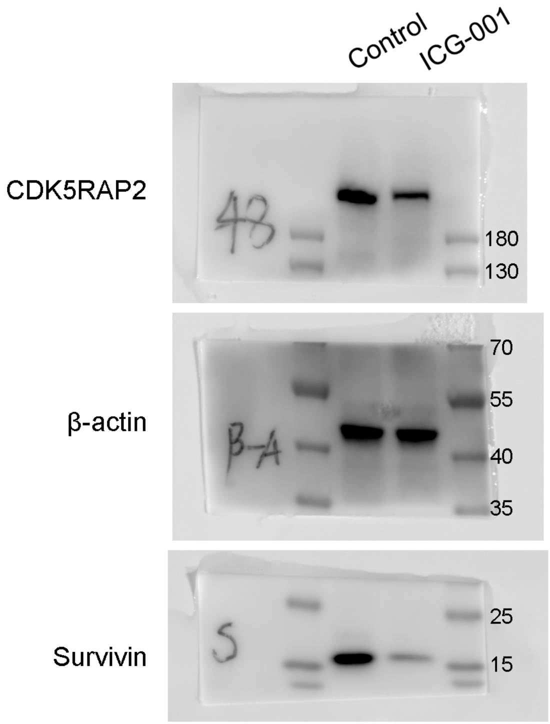

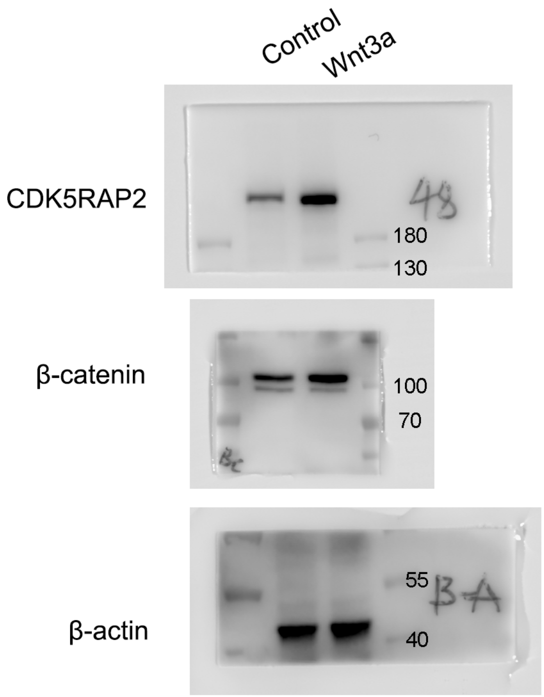

Original Western blots of Supplementary Figure 1

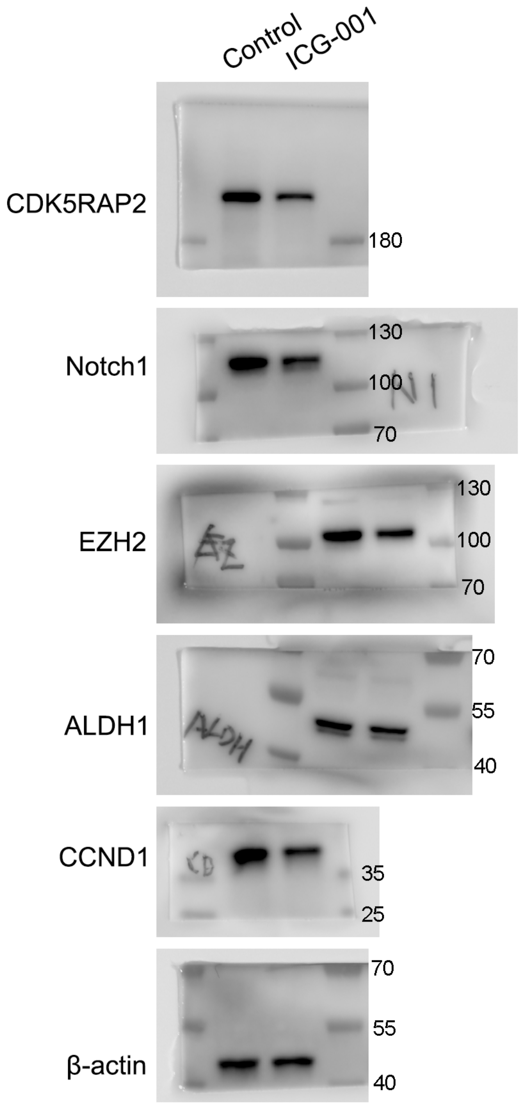

Original Western blots of Figure 3

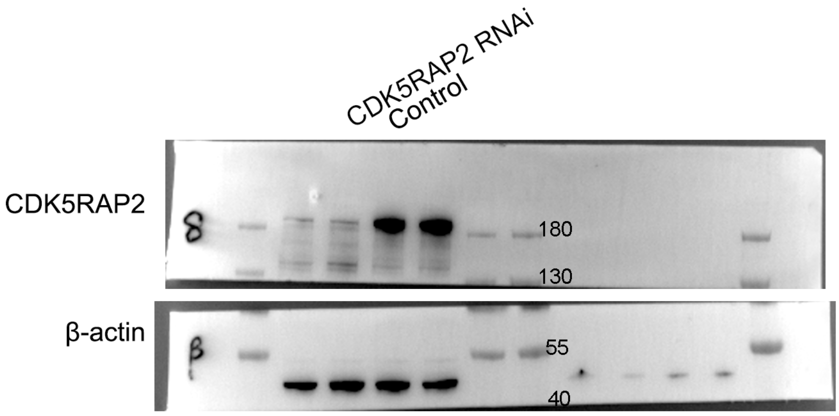

Original Western blots of Supplementary Figure 3

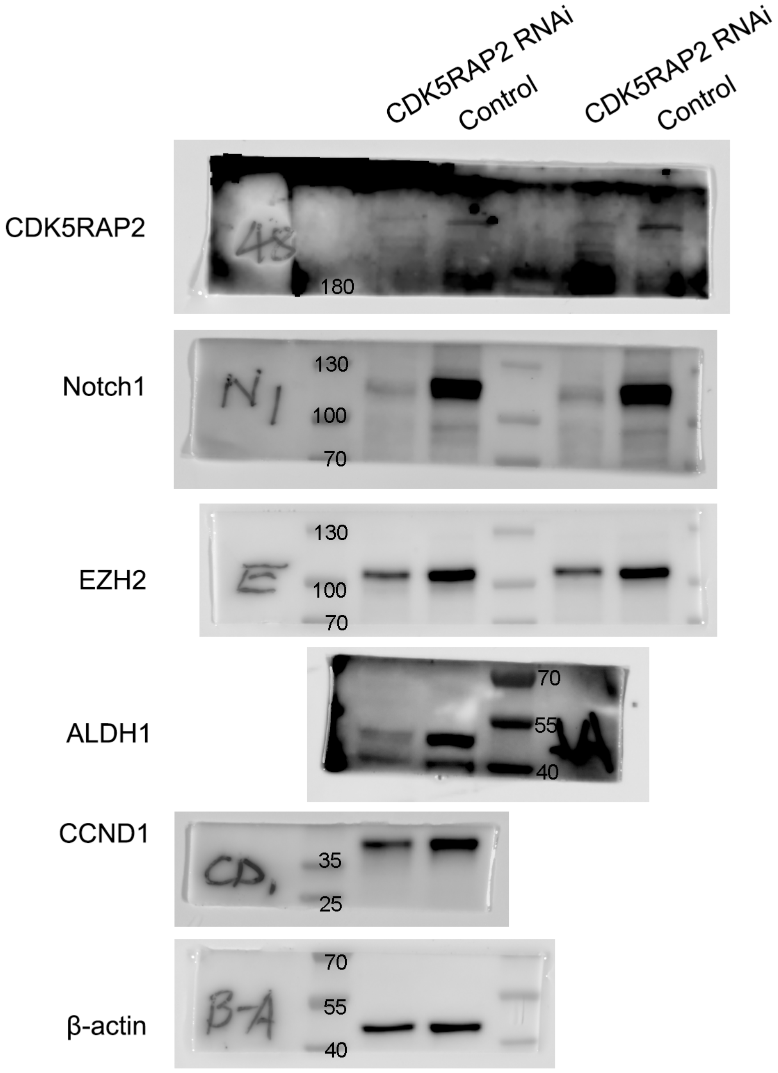

Supplement: Supplementary file 2 — Original Western blots of Figures [file 41419_2023_5652_MOESM2_ESM.pdf]
