# Supplementary material for: CDK5RAP2 is a Wnt target gene and promotes stemness and progression of oral squamous cell carcinoma
Source: Cell Death Dis. 2023 Feb 11;14(2):107. doi: 10.1038/s41419-023-05652-z (PMC9922250; doi:10.1038/s41419-023-05652-z)
Supplement: Supplementary file 3 — Author Contribution Statement [file 41419_2023_5652_MOESM3_ESM.pdf]

Author Contribution Statement

YS designed the experiments and drafted the manuscript. YS, YC, YL (Yuntao Lin), PL, BZ, and KCC conducted the experiments. YW contributed to the sample collection. YS, YC, and YL (Yicun Li) conducted statistical analysis. YS, RZQ and HY verified the underlying data. RZQ, HY, NKM, and MK revised the manuscript. All authors approved the final manuscript.
